# Gender-related differences in cardiometabolic risk factors and lifestyle behaviors in treatment-seeking adolescents with severe obesity

**DOI:** 10.1186/s12887-018-1057-3

**Published:** 2018-02-14

**Authors:** Lisa Ha Barstad, Pétur B. Júlíusson, Line Kristin Johnson, Jens Kristoffer Hertel, Samira Lekhal, Jøran Hjelmesæth

**Affiliations:** 1 Morbid Obesity Centre, Vestfold Hospital Trust, Box 2168, 3103 Tønsberg, Norway; 2 Department of Clinical Science, University of Bergen, 5021 Bergen, Norway; 3 Department of Endocrinology, Morbid Obesity and Preventive Medicine, Institute of Clinical Medicine, University of Oslo, Oslo, Norway

**Keywords:** Severe obesity, Gender differences, Lifestyle behavior, Metabolic syndrome, Cardiovascular risk factor, Cardiovascular disease

## Abstract

**Background:**

Obesity during adolescence is associated with cardiovascular mortality in adulthood. The adverse obesity-related cardiometabolic risk profile is already observed in adolescence. We aimed to examine possible gender differences in cardiometabolic risk factors and lifestyle behaviors among adolescents with severe obesity, hypothesizing that boys would have both a higher prevalence of the metabolic syndrome as well as less healthy lifestyle behaviors than girls.

**Methods:**

Cross-sectional study of treatment-seeking adolescents with severe obesity who attended the Morbid Obesity Centre at Vestfold Hospital Trust and who were consecutively enrolled in the Vestfold Register of Obese Children between September 2009 and September 2015. A total of 313 adolescents aged 12 to 18 years were recruited, whereof 268 subjects (49% boys) completed a food and activity frequency questionnaire and were included in the analysis.

**Results:**

Mean (SD) age, BMI and BMI SDS were 15 (1.6) years, 38.6 (5.9) kg/m^2^ and 3.5 (0.6). Levels of LDL cholesterol, fasting insulin and glucose and diastolic blood pressure (DBP) did not differ between genders. Compared to girls, boys had significantly higher triglycerides (*p* = 0.037) and systolic blood pressure (SBP) (*p* = 0.003), as well as lower HDL cholesterol (*p* = 0.002). The metabolic syndrome was present in 27% of the boys and 19% of the girls (*p* = 0.140), and the prevalence of high DBP, dyslipidemia and dysglycemia also did not differ significantly between genders. The prevalence of high SBP was higher in boys than in girls (19% vs. 9%, *p* = 0.021). Gender was associated with a number of lifestyle habits, as a larger proportions of boys had higher screen time (*p* = 0.032), more regular breakfast eating (*p* = 0.023), higher intake of sugar sweetened soda (*p* = 0.036), and lower intake of vegetables than girls (*p* = 0.011). By contrast, physical activity level and intake of fruit and berries did not differ between genders.

**Conclusions:**

Male treatment-seeking adolescents with severe obesity had a more unfavorable set of metabolic and behavioral risk factors for cardiovascular disease than girls. Our results indicate that lifestyle behavioral markers should be thoroughly assessed in both genders, and possible gender-related differences in risk profile should be taken into account in future treatment programs.

**Electronic supplementary material:**

The online version of this article (10.1186/s12887-018-1057-3) contains supplementary material, which is available to authorized users.

## Background

Cardiovascular diseases (CVD) are the leading cause of mortality in both men and women.

Importantly, men have a 3–4-fold higher risk of an acute coronary event below age 60 than women [1], although the prevalence of ischemic heart disease in younger women is increasing [2].

During the last 30 years, the prevalence of overweight and obesity in children has increased to alarming proportions in many Western countries, with obesity currently affecting one-fifth of US adolescents aged 12 to 19 years [3, 4]. Obesity during the teen years is not only a psychosocial burden, but is also related to increased mortality in middle age from CVD and shortened life expectancy [5, 6]. The adverse cardiometabolic risk profile related to obesity is observed in early adolescence and even in early childhood [7–11], and cardiometabolic risk factors are more prevalent with the increasing degree of obesity in adolescents, particularly in boys [9, 10, 12]. Furthermore, adolescent boys tend to have larger abdominal fat deposits than girls, a condition associated with hypertension, dyslipidemia and dysglycemia [13].

A number of studies have shown that unhealthy lifestyle behaviors such as low physical activity level, high screen time, skipping breakfast, high intake of sugar-sweetened beverages (SSB) and low intake of fruits and vegetables are related to obesity and cardiometabolic risk factors in children and adolescents, and with CVD in adults [14–23]*.* However, the causes and consequences of obesity in adolescence differ between the genders due to biological and behavioral differences [24–26]. Thus, there is a need for a clearer focus on gender-related differences within the field of adolescent obesity. Considering that adolescent girls may be more likely to modify their diet to obtain weight control than boys [27], and boys have a more disadvantageous fat distribution after puberty, an unhealthy lifestyle could have greater impact on future CVD risk in boys*.* Although some cross-sectional studies of CVD risk factors in adolescents with obesity have considered boys and girls separately, few studies have examined gender-related differences specifically [7, 9, 10, 12]. In addition, to the best of our knowledge, no study has examined gender-related differences in multiple lifestyle habits and cardiometabolic risk factors among adolescents with severe obesity.

We aimed to examine possible gender differences in lifestyle behaviors and cardiometabolic risk factors in a population of treatment seeking adolescents with severe obesity. We hypothesized that compared to girls, boys would have both a higher prevalence of the metabolic syndrome and its components as well as less healthy lifestyle behaviors. Finally, we aimed to assess the possible associations between cardiometabolic risk factors and lifestyle behaviors, hypothesizing that unhealthy lifestyle habits were associated with unfavorable levels of cardiometabolic risk factors.

## Methods

Treatment-seeking adolescents with severe obesity referred to a tertiary care obesity outpatient clinic (the Morbid Obesity Centre, Vestfold Hospital Trust (MOC)) in southern Norway and who were consecutively enrolled in the Vestfold Register of Obese Children from September 2009 until September 2015 were assessed for eligibility. The MOC receives patients who have severe obesity, referred by primary care practitioners. The main inclusion criterion is a BMI (body mass index) of 5 kg/m^2^ or more above the obesity limits proposed by the International Obesity Task Force (IOTF 30 kg/m^2^ (iso-BMI 30) + 5 kg/m^2^) [28], or a BMI level below this cut-off in the presence of obesity-related comorbidity (including family history of obesity-related comorbidity, type 2 diabetes mellitus, hypertension, dyslipidemia, very rapid increase in weight, severe psychosocial problems). We have defined severe obesity as BMI ≥ iso-BMI 35 as suggested by Bervoets et al. [29]. In addition to this definition we have also included subjects with BMI *≥* iso-BMI 30 and having obesity-related conditions. Of the 313 patients included in the Vestfold Register of Obese Children during this period, 268 subjects (86%) completed the food- and activity questionnaire on their first visit to the clinic and were included in this cross-sectional study.

### Food and activity frequency questionnaire

The participants’ lifestyle behaviors including physical activity, screen time, breakfast eating frequency and intake of sugar-sweetened soda, fruit and vegetables were reported using a self-administered food frequency questionnaire (FFQ) including questions about physical activity. The food and activity questionnaire included questions about habitual daily consumption of specified meals and 23 food items, daily physical activity outside school and daily screen time outside school (time in front of the TV- or PC screen). For each of the questions regarding screen time, physical activity level, breakfast eating, intake of fruit, vegetables and sugar-sweetened soda there were six to eight frequency categories to choose from as shown in Additional file 1: Table S1. The questionnaire was developed by the Department of Nutrition at the University of Oslo, Norway for use in a nationwide study of diet among fourth- and eighth-grade children (9- and 13 years old) in year 2000; the UNGKOST study [30]. The food frequency part of the questionnaire in the UNGKOST study has been validated against a four-day precoded food diary also used in the same study by Lillegaard and colleagues, and they demonstrated that the FFQ can be used to distinguish between low and high consumers of various food items [31]. Furthermore, they demonstrated that self-reported data from the FFQ were more consistent with data obtained from the food diary in the UNGKOST study considering intake of drinks, fruits and vegetables compared to infrequently eaten food items such as chocolate, sweets, savory snacks and pizza [31].

In the present analysis the highest frequencies of intake of fruits, vegetables and soda intake were rarely reported, therefore the number of original response categories were reduced and recoded into 3: low, moderate or high intake of fruits and berries and vegetables and low, moderate or high intake of sugar-sweetened soda. For simplicity, the response categories for physical activity level, screen time and breakfast eating were also reduced and recoded into 3: low, moderate or high physical activity level, low, moderate or high screen-time and skipping breakfast regularly, sometimes eating breakfast or regularly eating breakfast (Additional file 1: Table S1). Low physical activity, skipping breakfast regularly, high screen time, low intake of fruits and vegetables and/or high intake of sugar-sweetened soda were considered as unhealthy lifestyle behaviors and CVD risk factors.

### Anthropometric and clinical measurements

Height was measured using Heightronic Digital Stadiometer® to the nearest 0.1 cm. Weight (kg) and body fat percentage was measured using Tanita bioimpedance body composition analyzer (BC-418, 8-polar, TANITA Corp., Tokyo, Japan). Waist circumference (WC) was measured using a standardized anthropometric tape, measuring the circumference at the midpoint between the top of the iliac crest and the lower part of the lateral rib cage to the nearest 0.1 cm. Waist to height ratio (WHtR) was calculated as WC (in cm) divided by height (in cm). BMI was converted to SDS by means of the current Norwegian growth references [32].

Systolic and diastolic blood pressure (SBP and DBP) were measured using a digital oscillimetric device, Dinamap ProCare (GE Healthcare). BP measurements were taken in the sitting position using appropriate sized cuffs (the mid-upper arm circumference was measured) four times on the dominant arm, and the average between the last three measurements was calculated.

### Laboratory analyses

Blood samples were obtained after an overnight fast by venipuncture in vacutainer gel tubes, and serum was separated from cells by centrifugation. Analyses of serum glucose and blood lipids were performed using dry reagent slide technology on the Vitros FS 5.1 (Ortho-Clinical Diagnostics, NY, USA). HbA1c was analyzed by high-performance liquid chromatography (HLPC) using Tosoh HLC-723 G7 (Tosoh Corporation, Tokyo, Japan). Insulin was analyzed by radioimmunossay (RIA) (Millipore Corporation, Billerica, MA, USA) until March 2012, thereafter the electrochemiluminescence immunoassay method (ECLIA) (ECLIA kit, Roche Diagnostics, Mannheim, Germany) was used. Intra- and inter-assay coefficient of variation were < 10% for all assays. Insulin resistance was estimated using the homeostasis model assessment for insulin resistance (HOMA-IR): (insulin (pmol/L) x fasting blood glucose (mmol/L)/135) [33].

### Metabolic syndrome

Metabolic syndrome was identified using the definition from the International Diabetes Federation (IDF) Consensus Group Criteria: Abdominal (central) obesity (waist circumference ≥ 90th percentile for age and sex for ages < 16 years, and ≥ 94 cm for males and ≥80 cm for female for ages ≥ 16 years) and the presence of two or more of the following characteristics: 1) triglycerides ≥ 1.7 mmol/L, 2) HDL cholesterol < 1.03 mmol/L for age 10- < 16 years, and < 1.03 mmol/L in males and < 1.29 mmol/L in females for age ≥ 16 years, 3) SBP ≥ 130 mmHg or DBP ≥ 85 mmHg and 4) fasting plasma glucose ≥ 5.6 mmol/L or known type 2 diabetes [34]. Waist circumference was missing for 10 boys and 6 girls, and for these we assumed abdominal adiposity when BMI was above the International Obesity Task Force threshold for obesity [28].

### Ethics

All procedures in the study were performed in accordance with the ethical standards of the institutional and/or national research committee and with the Helsinki declaration and its later amendments or comparable ethical standards. The research protocol for the Vestfold Register of Obese Children has been approved by the Regional Committees for Medical and Health Research Ethics (S-08742c 2008/19081), Norwegian Centre for Research Data (NSD) and by the Norwegian Data Inspectorate (20,789 grh/rh). The research protocol for the current study has been approved by the Regional Committees for Medical and Health Research Ethics, REK sør-øst (2016/2039). Written informed consent was obtained from all participants and the parents/guardians of participants under the age of 16 in the study.

### Statistics

Continuous variables are presented as mean (SD) if normally distributed, or as median (interquartile range) if not. Categorical variables are presented as counts and percentages. Crude comparisons between variables were performed using Fisher’s exact test (categorical variables), independent samples t-test and ANOVA with Tukey’s post-hoc test (continuous normally distributed data), or Mann-Whitney Wilcoxon test or Kruskal-Wallis test (for continuous skewed data such as triglycerides, fasting glucose and insulin). The standardized mean differences (Cohen’s *d*) for cardiometabolic variables between the genders were calculated. The correlations between lifestyle habits (ordinal variables) and cardiometabolic markers (continuous variables) were analyzed with Spearman correlation. A probability value < 0.05 was considered statistically significant. Statistical analyses were performed with SPSS version 21 (Statistical Package for Social Science Inc., Chicago, IL, USA).

## Results

Data from a total of 268 (49% boys) treatment seeking adolescents with obesity aged 12 to 18 years were included in the analyses. Mean (SD) age, BMI and BMI SDS was 15.0 (1.6) years, 38.6 (5.9) kg/m^2^ and 3.5 (0.6) respectively. The boys were slightly younger than the girls (Table 1).

**Table 1:** Clinical characteristics of the study participants (*n* = 268) according to gender

	Boys (*n* = 132)	Girls (*n* = 136)	*P*-value
Age (years)	14.6 (1.7)	15.3 (1.5)	0.001
Weight (kg)	112.5 (24.8)	107.0 (18.6)	0.040
BMI (kg/m^2^)	37.9 (6.1)	39.2 (5.7)	0.075
BMI SDS	3.2 (0.4)	3.8 (0.6)	< 0.001
Delta iso-BMI 30	10.0 (5.6)	10.3 (5.6)	0.647
Waist circumference (cm)	115.0 (13.3)	109.3 (11.5)	< 0.001
Waist to height ratio	0.67 (0.07)	0.66 (0.07)	0.600
Body fat (%)	42.1 (7.3)	47.2 (5.1)	< 0.001
SBP (mmHg)	119 (15)	113 (14)	0.003
DBP (mmHg)	62 (7)	61 (7)	0.418
Total cholesterol (mmol/L)	4.4 (0.9)	4.5 (0.7)	0.458
HDL cholesterol (mmol/L)	1.1 (0.2)	1.2 (0.3)	0.002
LDL cholesterol (mmol/L)	2.6 (0.7)	2.6 (0.6)	0.505
Triglycerides (mmol/L)	1.4 (1.0–2.1)	1.3 (0.9–1.7)	0.037
Fasting insulin (pmol/L)	156 (112–236)	149 (109–202)	0.209
Fasting glucose (mmol/L)	4.9 (4.7–5.1)	4.9 (4.6–5.1)	0.133
HbA1c (%)	5.4 (0.6)	5.4 (0.5)	0.516
HOMA-IR^a^	5.8 (4.1–8.9)	5.3 (3.9–7.4)	0.270

Continuous variables are shown as mean (SD) or median (interquartile range) unless otherwise indicated. ^a^HOMA-IR = (insulin (pmol/L) x fasting blood glucose (mmol/L))/135

### Cardiometabolic risk factors

The cardiometabolic characteristics presented in Table 1 demonstrate that the mean waist circumference, triglyceride levels and SBP were significantly higher in boys compared with girls. In addition, boys had lower body fat percentage and HDL cholesterol levels than girls. The standardized mean differences between genders (Cohen’s *d*) are shown in Additional file 2: Table S2. Three of the participants (all boys) did not have central adiposity as defined by the IDF guidelines [34]. The prevalence of metabolic syndrome and components of the metabolic syndrome such as high DBP, dyslipidemia and dysglycemia did not differ significantly between genders (Table 2). However, the prevalence of high systolic blood pressure was higher in boys than in girls (19% vs. 9%, *p* = 0.021).

**Table 2:** Participants with metabolic syndrome and risk factors according to the IDF definition [34]

	Boys, n (%)	Girls, n (%)	*P*-value
Metabolic syndrome	34 (27)	25 (19)	0.140
High triglycerides	49 (39)	36 (27)	0.064
Low HDL cholesterol	49 (39)	48 (36)	0.702
High systolic blood pressure	25 (19)	12 (9)	0.021
High diastolic blood pressure	0	0	
High fasting glucose	3 (2)	8 (6)	0.218

Fisher’s exact test

### Unhealthy lifestyle behaviors

**Fig. 1:**
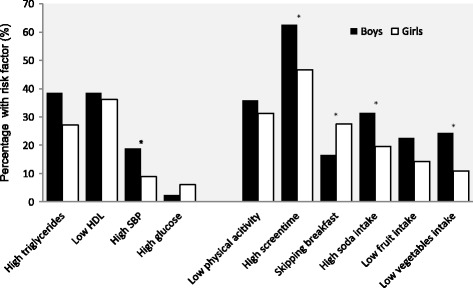
Prevalence of CVD risk factors (cardiometabolic and behavioral) according to gender. *P < 0.05

Gender was significantly associated with a number of lifestyle habits, as larger proportions of boys had higher screen time, more frequent breakfast eating, higher intake of sugar sweetened soda, and lower intake of vegetables than girls (Table 3). By contrast, physical activity level and intake of fruit and berries did not differ between boys and girls. The percentages of all adolescents reporting unhealthy lifestyle habits were 34% for low physical activity level, 55% for high screen time, 28% for not eating breakfast regularly, 25% for high intake of sugar-sweetened soda, and 18% for low intake of vegetables, fruits and berries. In a sub-analysis, those with unhealthy lifestyle behaviors (low physical activity, skipping breakfast regularly, high screen time, low intake of fruits and vegetables and/or high intake of sugar-sweetened soda) were compared with those reporting healthier lifestyle behaviors. As compared with girls, a larger proportion of boys reported high screen time (*p* = 0.010) and high intake of sugar-sweetened soda (*p* = 0.041). By contrast, the percentage of girls skipping breakfast regularly was higher than for boys (*p* = 0.037). There were no significant differences in the proportions of boys and girls with low physical activity (*p* = 0.436) or low intake of fruit (*p* = 0.223) and vegetables (*p* = 0.133). Figure 1 shows the prevalence of CVD risk factors (both cardiometabolic and lifestyle habits) according to gender.

**Table 3:** Physical activity level, screen time, breakfast eating frequency, intake of sugar-sweetened soda, fruits and vegetables

	Boys, n (%)	Girls, n (%)	*P*-value
Physical activity level			0.704
Low	46 (36)	42 (31)	
Moderate	72 (56)	82 (61)	
High	10 (8)	10 (8)	
Screen time			0.032
Low	11 (8)	15 (11)	
Moderate	38 (29)	57 (42)	
High	82 (63)	63 (47)	
Breakfast eating			0.023
Skipping regularly	21 (17)	36 (28)	
Sometimes	25 (20)	33 (25)	
Regularly	81 (64)	62 (47)	
Sugar-sweetened soda			0.036
Low	27 (22)	44 (34)	
Moderate	56 (46)	59 (46)	
High	38 (31)	25 (20)	
Fruits and berries			0.179
Low	28 (23)	18 (14)	
Moderate	62 (50)	65 (51)	
High	34 (27)	44 (35)	
*Vegetables*			0.011
Low	31 (24)	14 (11)	
Moderate	73 (58)	81 (63)	
High	23 (18)	34 (26)	

Fisher’s exact test

### Associations between cardiometabolic markers and lifestyle behaviors

The associations between lifestyle behaviors (physical activity level, breakfast eating frequency, frequency of intake of fruits, vegetables and sugar-sweetened soda) and cardiometabolic markers (total cholesterol, HDL-and LDL cholesterol, waist circumference, triglycerides, glucose, SBP and DBP) were significant only in terms of screen time and DBP, *r* = 0.17, *p* = 0.005). In the girl cohort HDL cholesterol was significantly related to breakfast intake frequency (*r* = 0.21, *p* = 0.020), whilst in boys DBP was related to screen time and fruit intake frequency (*r* = 0.26, *p* = 0.002, and *r* = − 0.22, *p* = 0.016, respectively). The mean levels of HDL cholesterol, LDL cholesterol, triglycerides, SBP and DBP did not differ significantly between those with unhealthy and healthier lifestyle behaviors (Tables 4 and 5). However, fasting blood sugar was significantly higher in those with high physical activity compared with those with low physical activity (Table 4).

**Table 4:** Cardiometabolic risk markers at different levels of breakfast eating, physical activity and screen time

	Physical activity level			Breakfast eating			Screen time		
Cardiometabolic risk marker	Low	Moderate	High	Skipping regularly	Sometimes	Regular	Low	Moderate	High
	*n* = 88	*n* = 154	*n* = 20	*n* = 57	*n* = 58	*n* = 143	*n* = 26	*n* = 95	*n* = 145
HDL cholesterol (mmol/L)	1.19 (0.27)	1.19 (0.26)	1.25 (0.33)	1.17 (0.24)	1.17 (0.26)	1.19 (0.28)	1.20 (0.27)	1.22 (0.27)	1.17 (0.28)
LDL cholesterol (mmol/L)	2.6 (0.6)	2.6 (0.7)	2.6 (0.7)	2.5 (0.6)	2.6 (0.7)	2.6 (0.7)	2.7 (0.7)	2.5 (0.7)	2.6 (0.7)
Triglycerides (mmol/L)	1.5 (0.8)	1.5 (0.8)	1.6 (0.9)	1.5 (0.6)	1.5 (0.7)	1.6 (0.9)	1.4 (0.6)	1.5 (0.7)	1.6 (0.8)
Fasting glucose (mmol/L)	4.9 (0.3)^a^	4.9 (0.3)	5.4 (2.0)^1^	4.9 (0.4)	4.9 (0.4)	5.0 (0.9)	4.9 (0.4)	4.9 (0.4)	4.9 (0.9)
Systolic blood pressure (mmHg)	118 (11)	115 (16)	119 (15)	116 (12)	112 (21)^a^	118 (12)^a^	111 (22)	115 (11)	118 (15)

ANOVA with post-hoc test for HDL cholesterol, LDL cholesterol and systolic blood pressure. Kruskall-Wallis test for triglycerides and fasting glucose. ^a^Values within the same lifestyle category differ significantly from each other

**Table 5:** Cardiometabolic risk markers at different levels of intake of sugar-sweetened soda, fruits/berries and vegetables

	Sugar-sweetened soda			Fruits and berries			Vegetables		
Cardiometabolic risk marker	Low	Moderate	High	Low	Moderate	High	Low	Moderate	High
	*n* = 70	*n* = 109	*n* = 62	*n* = 45	*n* = 122	*n* = 76	*n* = 42	*n* = 150	*n* = 56
HDL cholesterol (mmol/L)	1.14 (0.27)	1.20 (0.28)	1.19 (0.25)	1.21 (0.27)	1.20 (0.28)	1.16 (0.25)	1.20 (0.31)	1.20 (0.28)	1.15 (0.23)
LDL cholesterol (mmol/L)	2.6 (0.7)	2.6 (0.7)	2.6 (0.7)	2.7 (0.6)	2.5 (0.7)	2.6 (0.8)	2.7 (0.7)	2.5 (0.7)	2.6 (0.8)
Triglycerides (mmol/L)	1.4 (0.7)	1.6 (0.9)	1.5 (0.7)	1.5 (0.6)	1.5 (0.8)	1.6 (0.8)	1.5 (0.6)	1.5 (0.8)	1.4 (0.8)
Fasting glucose (mmol/L)	5.0 (1.1)	4.9 (0.5)	4.9 (0.4)	5.0 (0.6)	4.9 (0.9)	4.9 (0.4)	4.9 (0.4)	4.9 (0.8)	4.9 (0.5)
Systolic blood pressure (mmHg)	115 (11)	116 (15)	116 (18)	111 (24)	117 (11)	116 (12)	116 (11)	116 (15)	116 (17)

ANOVA with post-hoc test for HDL cholesterol, LDL cholesterol and systolic blood pressure. Kruskall-Wallis test for triglycerides and fasting glucose

## Discussion

In accordance with our hypothesis, we found that treatment seeking adolescent boys with obesity had a significantly worse cardiometabolic risk profile than girls, including unfavorable measures of waist circumference, blood pressure, triglycerides and HDL cholesterol. In addition, larger proportions of boys had unhealthy lifestyle behaviors such as higher screen time, higher intake of sugar-sweetened soda and lower intakes of vegetables than girls. However, in contrast with our hypothesis, the prevalence of metabolic syndrome did not differ significantly between genders, and a higher proportion of girls than boys skipped breakfast regularly. Finally, most cardiometabolic risk factors were not significantly associated with lifestyle behaviors.

### Cardiometabolic risk factors

One out of four boys (27%) and one out of five girls (19%) had metabolic syndrome. These figures are in accordance with those from an Italian cohort of adolescents with severe obesity (30 and 19%, respectively), but lower than demonstrated in a German cohort (46 and 37%, respectively) [12]. Although in the current study the average serum triglyceride levels were higher and HDL cholesterol levels lower in boys than in girls, the percentage with high triglycerides or low HDL cholesterol did not differ significantly between genders. Furthermore, high blood pressure was diagnosed in 19% of the boys, which was significantly higher than in the girls (9%). In two other cross-sectional studies prehypertension was present in 25% of adolescents with obesity and hypertension was present in 5%, but these studies did not assess gender differences [9, 10]. However, we did not find significant differences in cardiometabolic risk factors between adolescents with unhealthy or healthier lifestyle behaviors, except for blood glucose which was higher in those with high physical activity. Thus, we could not confirm our hypothesis that unhealthy lifestyle habits were associated with more unfavorable levels of cardiometabolic risk factors in our cohort of adolescents with severe obesity.

### Lifestyle behaviors

In the current study, 55, 34 and 22% of treatment seeking adolescents with severe obesity had high screen time, low physical activity and skipped breakfast regularly. These figures are considerably higher than those from a representative population of Norwegian 13-year olds taken from a national food and activity survey; the corresponding percentages were 18, 10 and 10% [30]. In the current study more boys than girls had high screen time as well as high intake of sugar-sweetened beverages. High screen time has been associated with high total cholesterol to HDL cholesterol ratio, high triglycerides and obesity in children [15, 17, 35]. In addition, Stamatakis et al. showed that screen time of 4 h or more per day in adults doubled the risk of CVD (HR:2.25 (95% CI:1.30–3.89)) [20]. A study by Shang et al. found a higher energy intake of 136 kcal and lower intake of fruits and vegetables of 0.2 servings in overweight children with high screen time in comparison with those with low screen time [36]. Data from the first National Health and Nutrition Examination Survey Epidemiologic Follow-up Study showed that adults who reported a high intake of fruits and vegetables had a 27% lower CVD mortality than those who had a low intake of fruits and vegetables [22]. Furthermore, sugar-sweetened beverages have been associated with weight gain, metabolic syndrome and type 2 diabetes [23, 37].

Furthermore, in the current study, a high proportion of boys and girls did not eat breakfast regularly, although the percentage of girls skipping breakfast regularly was higher than among boys. This is in accordance with findings of Boutelle et al. who suggest that girls may skip meals more often than boys as a method of losing weight [38]. Not eating breakfast regularly has been associated with increased waist circumference, high fasting insulin, high glucose and obesity in both children and adolescents [19, 39]. Skipping breakfast is associated with a poor diet quality, with low intake of whole-grain products and low micronutrient intake [18, 40].

### Strengths and limitations

One strength of this study is the relatively large cohort of treatment-seeking adolescents with severe obesity. Furthermore, in the Vestfold Register of Obese Children a wide range of cardiometabolic markers with clinical relevance have been registered. There are, however, limitations to our study which need to be addressed. The questionnaire was based on self-reported data about physical activity and dietary habits. In a population classified as obese, under-reporting of unhealthy choices and over-reporting of healthy choices can be expected [41].

The apparent lack of association between lifestyle habits and cardiometabolic risk factors might be explained by either the relatively low number of participants in the extreme categories (lowest, highest), or that severe adiposity has a larger impact on cardiometabolic risk factors than lifestyle behavior.

## Conclusion

Treatment-seeking adolescent boys with severe obesity had a more unfavorable cardiometabolic risk profile, higher screen time, higher intake of sugar-sweetened soda and lower intake of vegetables than girls, while a higher proportion of girls skipped breakfast regularly. In a clinical setting a thorough assessment of health-related factors including lifestyle behavioral markers should receive proper attention to be able to identify relevant treatment focus and individualize the treatment in both genders. Care providers should, however, be aware of the possibility of differences in risk profiles between boys and girls.

## Additional files

Additional file 1: **Table S1.** Original response categories in the food and activity questionnaire and new response categories after recoding. (DOCX 14 kb)

Additional file 2: **Table S2.** Absolute and standardized mean differences (Cohen’s *d*) for cardiometabolic variables for boys compared to girls. (DOCX 14 kb)

## Abbreviations

CVD: Cardiovascular disease
FFQ: Food frequency questionnaire
IDF: International Diabetes Federation
MOC: Morbid Obese Centre, Vestfold Hospital Trust
SSB: Sugar-sweetened beverages
WC: Waist circumference
WHtR: Waist to height ratio
