# Supplementary material for: Gender-related differences in cardiometabolic risk factors and lifestyle behaviors in treatment-seeking adolescents with severe obesity
Source: BMC Pediatr. 2018 Feb 14;18:61. doi: 10.1186/s12887-018-1057-3 (PMC5813385; doi:10.1186/s12887-018-1057-3)
Supplement: Supplementary file 1 — Table S1. Original response categories in the food and activity questionnaire and new response categories after recoding. (DOCX 14 kb) [file 12887_2018_1057_MOESM1_ESM.docx]

Supplementary Table 1. Original response categories in the food and activity questionnaire and new response categories after recoding.

| Question | Original response categories | Recoded into |
| --- | --- | --- |
| Physical activity level | Never  Less than once per month  1-3 times per month | Low: 0-3 times per month |
|  | 1 time per week  2-3 times per week | Moderate: 1-3 times per week |
|  | 4-6 times per week  Every day | High: 4-7 times per week |
| Screen time | None  Less than 30 minutes per day  30 minutes-1 hour per day | Low: 1 hour or less per day |
|  | 2-3 hours per day | Moderate: 2-3 hours per day |
|  | 4 hours per day  More than 4 hours per day | High: 4 hours or more per day |
| Breakfast eating | Never or seldom  Once a week  Twice a week | Skipping regularly: 0-2 times per week |
|  | 3 times per week  4 times per week  5 times per week | Sometimes: 3-5 times per week |
|  | 6 times per week  Every day | Regularly: 6-7 times per week |
| Intake of sugar-sweetened soda | Never or seldom | Low: Never or seldom |
|  | 1-3 glasses per month  1-3 glasses per week | Moderate: At least one glass per month to 3 glasses per week |
|  | 4-6 glasses per week  1-3 glasses per day  4-6 glasses per day  7 glasses or more per day | High: At least 4 glasses per week |
| Intake of fruits and berries | Never or seldom  1-3 times per month | Low: A maximum of 3 times per month |
|  | 1-3 times per week  4-6 times per week | Moderate: 1-6 times per week |
|  | 1 time per day  2 times per day  3 times per day  4 times or more per day | High: At least once per day |
| Intake of vegetables | Never-seldom  1-3 times per month | Low: A maximum of 3 times per month |
|  | 1-3 times per week  4-6 times per week | Moderate: 1-6 times per week |
|  | 1 time per day  2 times per day  3 times per day  4 times or more per day | High: At least once per day |
