# Supplementary material for: Gender-related differences in cardiometabolic risk factors and lifestyle behaviors in treatment-seeking adolescents with severe obesity
Source: BMC Pediatr. 2018 Feb 14;18:61. doi: 10.1186/s12887-018-1057-3 (PMC5813385; doi:10.1186/s12887-018-1057-3)
Supplement: Supplementary file 2 — Table S2. Absolute and standardized mean differences (Cohen’s d) for cardiometabolic variables for boys compared to girls. (DOCX 14 kb) [file 12887_2018_1057_MOESM2_ESM.docx]

Supplementary Table 2. Absolute and standardized mean differences (Cohen’s *d*) for cardiometabolic variables for boys compared to girls.

|  | Absolute mean difference (95 % CI) | Standardized mean difference  (Cohen’s *d*) | Standardized mean difference^1^ |
| --- | --- | --- | --- |
| Waist circumference (cm) | 5.6 (2.5, 8.7) | 0.46 | S |
| Waist to height ratio | 0.005 (-0.012, 0.021) | 0 | T |
| Body fat (%) | -5.1 (-6.6, -3.6) | -0.84 | L |
| Systolic blood pressure (mmHg) | 5.3 (1.9, 8.7) | 0.37 | S |
| Diastolic blood pressure (mmHg) | 0.7 (-1.0, 2.3) | 0.09 | T |
| Total cholesterol (mmol/L) | -0.07 (-0.27, 0.12) | -0.09 | T |
| HDL cholesterol (mmol/L) | -0.11 (-0.17, -0.04) | -0.40 | S |
| LDL cholesterol (mmol/L) | -0.06 (-0.22, 0.11) | 0 | T |
| Triglycerides (mmol/L) | 0.24 (0.05, 0.42) | 0.27 | S |
| Fasting insulin (pmol/L) | 29.9 (2.0, 57.8) | 0.27 | S |
| Fasting glucose (mmol/L) | -0.03 (-0.20, 1.14) | -0.05 | T |
| HbA1c (%) | -0.04 (-0.17, 0.09) | 0 | T |
| HOMA-IR^1^ | 1.12 (0.07, 2.17) | 0.27 | S |

^1^T (Trivial) < 0.20, S (small) 0.20-0.49, M (medium) 0.50-0.79, L (large) ≥ 0.80
